# Clinical, Dermoscopic, and Histopathological Correlation of Pigmented Skin Lesions at a Tertiary Care Dermatology Center

**DOI:** 10.7759/cureus.116571

**Published:** 2026-09-20

**Authors:** Rabia A Ghafoor Sadal, Misbah Salim, Maryam Qayyum, Anam Gul

**Affiliations:** 1 Department of Dermatology, CMH Kharian Medical College, Gujrat, PAK; 2 Department of Dermatology, Maryam Hospital, Gujrat, PAK; 3 Department of Dermatology, DHQ Hospital Jhelum, Jhelum, PAK

**Keywords:** cutaneous malignancy, dermoscopy, diagnostic accuracy, histopathology, melanoma, pigmented skin lesions

## Abstract

Background and objective

Pigmented skin lesions encompass a wide spectrum of benign and malignant conditions that may present with overlapping clinical features. Dermoscopy enhances visualization of subsurface structures and can improve early detection of cutaneous malignancies when correlated with histopathological findings. However, local evidence on the agreement and diagnostic performance of clinical examination and dermoscopy compared to histopathology remains limited, particularly within tertiary care dermatology settings in Pakistan. This study was conducted to evaluate the clinical, dermoscopic, and histopathological correlation of pigmented skin lesions and to determine the diagnostic accuracy of dermoscopy in detecting cutaneous malignancies.

Methodology

This prospective observational study was carried out at Combined Military Hospital (CMH) Kharian Medical College, Gujrat, Pakistan, from January 2024 to June 2024 and enrolled 75 patients with pigmented skin lesions. All participants underwent a standardized clinical examination, dermoscopic evaluation, and biopsy with histopathological assessment. Patients with previously treated malignancies, non-evaluable lesions, contraindications to biopsy, or incomplete data were excluded.

Results

The mean age of the patients was 46.8 ± 15.2 years, and 41 (54.7%) were male. Histopathology confirmed 50 (66.7%) benign and 25 (33.3%) malignant lesions. Benign melanocytic nevus was the most common diagnosis, occurring in 25 (33.3%) patients, whereas basal cell carcinoma was the most frequent malignancy in 11 (14.7%), followed by malignant melanoma in eight (10.7%) and squamous cell carcinoma in six (8.0%). Malignant lesions had a significantly larger mean diameter than benign lesions (13.7 ± 4.1 mm vs. 7.8 ± 2.6 mm; p < 0.001). Border irregularity, color variegation, asymmetry, blue-white veil, atypical vascular patterns, and multiple dermoscopic colors were significantly more frequent in malignant lesions. Dermoscopy demonstrated a sensitivity of 92.0%, a specificity of 94.0%, a positive predictive value (PPV) of 88.5%, a negative predictive value (NPV) of 95.9%, and an overall diagnostic accuracy of 93.3%. Agreement with histopathology was stronger for dermoscopy than for clinical examination, with Cohen's kappa values of 0.84 and 0.62, respectively.

Conclusions

Dermoscopy demonstrated higher diagnostic performance and stronger agreement with histopathology than routine clinical examination. Its use may facilitate more accurate assessment and earlier identification of malignant pigmented skin lesions.

## Introduction

Pigmented skin lesions encompass a wide spectrum of disorders that include both melanocytic and non-melanocytic lesions, ranging from benign nevi and seborrheic keratoses to premalignant lesions and invasive cutaneous malignancies [1]. Pigmented skin lesions comprise a heterogeneous group of melanocytic and non-melanocytic conditions. Melanocytic lesions include benign melanocytic nevi, dysplastic nevi, and malignant melanoma, whereas non-melanocytic pigmented lesions may include seborrheic keratosis, pigmented basal cell carcinoma, squamous cell carcinoma, dermatofibroma, and certain vascular lesions. In recent decades, the incidence of skin cancers has grown worldwide due to the aging population, rising exposure to ultraviolet radiation (UVR), environmental factors, and greater public awareness of skin cancer [2]. The vast majority of these cancers are classified as non-melanoma skin cancers (NMSC) and include basal cell and squamous cell carcinomas, though the latter is responsible for the majority of skin cancer-related deaths because of its high potential for metastasis [2].

The first step in assessing pigmented skin lesions is a clinical examination, which may include the use of ABCDE criteria. These criteria look at asymmetry, border irregularity, color variation, diameter, and evolution of lesions; however, the use of these criteria may have limited diagnostic value, particularly for early melanomas and atypical benign lesions with similar features [3,4]. Given the diagnostic challenges posed by early melanomas and atypical pigmented lesions, evaluating clinicodermoscopic-histopathological concordance remains essential for improving diagnostic accuracy and supporting timely identification of cutaneous malignancies [5].

Dermoscopy is a non-invasive imaging technique that uses magnification and polarized or non-polarized light to visualize subsurface structures of the epidermis and superficial dermis. It helps in characterizing pigment networks, vascular patterns, dots, globules, and regression structures, and can also guide biopsy site selection and monitoring of suspicious lesions [6]. Dermoscopy is particularly useful for increasing the diagnostic value of pigment networks, vascular patterns, globules, streaks, and other dermoscopic features, thus enhancing the diagnostic accuracy of melanocytic and non-melanocytic skin lesions [7,8]. Many studies have shown that dermoscopy improves the sensitivity and specificity of diagnosis and helps avoid unnecessary treatment of non-cancerous lesions suspected of being melanoma [9]. The technique has also helped differentiate pigmented basal cell carcinoma, seborrheic keratosis, dermatofibroma, and vascular lesions based on their characteristic dermoscopic patterns [10,11].

Although clinical examination and dermoscopy provide considerable assistance in the diagnosis of pigmented skin lesions, definitive diagnosis is still achieved through histopathological examination [12]. Histopathology allows detailed evaluation of cellular morphology, architectural features, tumour thickness, mitoses, ulceration, and other features that help guide treatment planning and follow-up care for the patient [13]. Integration of clinical and dermoscopic information with histopathological assessment may improve diagnostic interpretation and agreement in melanocytic lesions [14]. Several studies have shown a high degree of concordance between dermoscopic and histopathological diagnoses, but discrepancies still exist, especially in atypical melanocytic proliferations and early melanomas [15]. It is therefore crucial to assess the level of agreement between clinical examination, dermoscopy, and histopathology in order to improve diagnostic accuracy and decrease the likelihood of delayed diagnosis and unnecessary biopsies [16]. The value of such evidence is especially important in tertiary centers where patients often present with clinically challenging or atypical pigmented lesions.

## Materials and methods

This prospective observational study was carried out at Combined Military Hospital (CMH) Kharian Medical College, Gujrat, Pakistan, from January 2024 to June 2024 and enrolled 75 patients presenting with clinically pigmented skin lesions. The study aimed to evaluate the clinical, dermoscopic, and histopathological correlation of pigmented skin lesions for the early detection of cutaneous malignancies. It was conducted after obtaining approval from the Institutional Ethical Review Committee of CMH Kharian Medical College. Written informed consent was obtained from all participants before enrollment, clinical examination, dermoscopic assessment, and biopsy. The study procedures were performed in accordance with institutional ethical standards and the principles of the Declaration of Helsinki.

The sample size was calculated to estimate the diagnostic sensitivity of dermoscopy in detecting cutaneous malignancies, using an anticipated sensitivity of 90%, an expected prevalence of malignant pigmented lesions of 33.3%, a 95% confidence level, and an absolute precision of 12%. The minimum required sample size was 73 patients. To account for incomplete or non-evaluable observations, 75 patients were included.

The calculation was based on the following formula:

*n = [Z&sup2; &times; Se &times; (1 &minus; Se)] / (d&sup2; &times; P)*

Where n = required sample size, Z = 1.96 for a 95% confidence level, Se = anticipated sensitivity of dermoscopy, P = expected prevalence of malignant pigmented lesions, and d = desired absolute precision

Patients aged 18 years or above, of either gender, presenting with one or more pigmented skin lesions suspicious for melanocytic or non-melanocytic pathology, and willing to undergo clinical examination, dermoscopic evaluation, and skin biopsy with histopathological examination, were included. Patients with previously diagnosed or treated skin malignancies, recurrent lesions, lesions with secondary infection or ulceration that precluded adequate dermoscopic examination, bleeding disorders that contraindicated biopsy, patients receiving chemotherapy or radiotherapy for skin cancer, individuals unwilling to undergo biopsy, and those with incomplete clinical or histopathological data were excluded.

Data collection

After obtaining approval from the Institutional Ethical Review Committee and written informed consent, eligible patients were enrolled using a non-probability consecutive sampling technique. Demographic and clinical information, including age, gender, duration of the lesion, family history of skin cancer, history of excessive sun exposure, Fitzpatrick skin type [17], lesion location, lesion size, color variation, border characteristics, surface changes, ulceration, bleeding, and evolution of the lesion, were recorded. Clinical examination was performed first, followed by dermoscopic assessment. One index pigmented skin lesion from each participant was included in the analysis. In patients who presented with multiple lesions, the clinically most suspicious lesion for malignancy was chosen as the index lesion. As a result, the final analysis included 75 index lesions from 75 patients.

Dermoscopic evaluation with a handheld polarized dermoscope followed the clinical examination. On clinical examination, lesions were categorized as benign, suspicious, or malignant according to morphological features, including asymmetry, border irregularity, color variation, diameter, evolution, ulceration, bleeding, and other clinically concerning features. Lesions with a strong clinical impression of malignancy were classified as malignant, lesions without suspicious clinical features were classified as benign, and lesions with one or more worrying but non-definitive features were classified as suspicious. Pigment network, asymmetry, blue-white veil, atypical dots or globules, streaks, regression structures, atypical vascular patterns, multiple colors, arborizing vessels, milia-like cysts, and comedo-like openings were all evaluated during dermoscopic examination. Lesions without malignant dermoscopic features were classified as benign, lesions with equivocal or atypical features were classified as suspicious, and lesions showing characteristic dermoscopic features strongly suggestive of malignancy were classified as malignant.

The dermoscopic examiner was not blinded to the clinical appearance, since dermoscopy was performed after clinical examination. However, clinical and dermoscopic classifications were recorded before histopathological results were available. An excisional, incisional, or punch biopsy was subsequently performed according to the lesion's characteristics and anatomical location. An experienced dermatopathologist, blinded to the dermoscopic diagnosis, examined tissue samples that had been fixed in 10% formalin. Histopathology was considered the reference standard for final diagnosis and included benign melanocytic nevus, dysplastic nevus, seborrheic keratosis, basal cell carcinoma, squamous cell carcinoma, malignant melanoma, and other pigmented lesions, where applicable.

The primary outcome was the degree of correlation among clinical diagnosis, dermoscopic diagnosis, and histopathological findings. Secondary outcomes included the frequency of benign and malignant pigmented skin lesions, the diagnostic accuracy of clinical examination and dermoscopy, sensitivity, specificity, positive predictive value, negative predictive value, and the proportion of histopathologically confirmed cutaneous malignancies detected through clinical and dermoscopic assessment.

Statistical analysis

Data were entered and analyzed using IBM SPSS Statistics for Windows, Version 26.0 (IBM Corp., Armonk, NY). Continuous variables were expressed as mean ± standard deviation (SD), while categorical variables were presented as frequencies and percentages. On the basis of clinical and dermoscopic assessment, lesions were initially categorized as benign, suspicious, or malignant. For the diagnostic accuracy analysis, these categories were dichotomized: lesions classified as suspicious or malignant were considered test-positive, while lesions classified as benign were considered test-negative. Histopathological diagnosis was dichotomized as benign or malignant and served as the reference standard. Sensitivity, specificity, positive predictive value (PPV), negative predictive value (NPV), and overall diagnostic accuracy were then calculated from the resulting 2 × 2 contingency tables. The Chi-square test or Fisher's exact test was used to compare categorical variables, and a p-value ≤ 0.05 was considered statistically significant.

## Results

The study included 75 patients with a mean age of 46.8 ± 15.2 years. The largest age group was 46-60 years, comprising 24 (32.0%) patients, followed by the 31-45 years group with 20 (26.7%), those older than 60 years with 16 (21.3%), and the 18-30 years group with 15 (20.0%). There were 41 (54.7%) males and 34 (45.3%) females. The mean duration of the lesions was 13.6 ± 8.9 months, while the mean lesion diameter was 9.8 ± 4.3 mm. Excessive sun exposure was reported by 39 (52.0%) patients, whereas nine (12.0%) had a family history of skin cancer (Table 1).

**Table 1:** Demographic and baseline clinical characteristics of patients with pigmented skin lesions (n = 75) SD: standard deviation

Characteristic	Category	Values
Age, years, mean ± SD		46.8 ± 15.2
Age group, years, n (%)	18–30	15 (20.0)
	31–45	20 (26.7)
	46–60	24 (32.0)
	> 60	16 (21.3)
Gender, n (%)	Male	41 (54.7)
	Female	34 (45.3)
Duration of lesion, months, mean ± SD		13.6 ± 8.9
Lesion diameter, mm, mean ± SD		9.8 ± 4.3
Occupational/excessive sun exposure, n (%)	Yes	39 (52.0)
	No	36 (48.0)
Family history of skin cancer, n (%)	Yes	9 (12.0)
	No	66 (88.0)

Malignant lesions had a significantly larger mean diameter than benign lesions, measuring 13.7 ± 4.1 mm compared to 7.8 ± 2.6 mm (p < 0.001). Border irregularity was observed in 19 (76.0%) malignant lesions and 13 (26.0%) benign lesions, while color variegation was present in 18 (72.0%) and 12 (24.0%), respectively. Asymmetry was identified in 17 (68.0%) malignant lesions compared to 11 (22.0%) benign lesions. Blue-white veil and atypical vascular pattern were each present in 13 (52.0%) malignant lesions, compared to two (4.0%) and five (10.0%) benign lesions, respectively. Multiple dermoscopic colors were observed in 14 (56.0%) malignant lesions compared to eight (16.0%) benign lesions, a difference that was statistically significant (χ² = 12.86, p < 0.001). All differences were statistically significant, with p-values < 0.001, except for multiple dermoscopic colors, which had a p-value of 0.001 (Table 2).

**Table 2:** Clinical and dermoscopic findings according to histopathological diagnosis The independent-samples t-test was used to compare mean lesion diameter between benign and malignant lesions. Pearson’s Chi-square test was used to compare categorical clinical and dermoscopic findings. A two-sided p-value < 0.05 was considered statistically significant SD: standard deviation

Variable	Benign lesions (n = 50)	Malignant lesions (n = 25)	Test statistic	P-value
Lesion diameter, mm, mean ± SD	7.8 ± 2.6	13.7 ± 4.1	t = −6.57	< 0.001
Border irregularity, n (%)	13 (26.0)	19 (76.0)	χ² = 17.03	< 0.001
Color variegation, n (%)	12 (24.0)	18 (72.0)	χ² = 16.00	< 0.001
Asymmetry, n (%)	11 (22.0)	17 (68.0)	χ² = 15.07	< 0.001
Blue-white veil, n (%)	2 (4.0)	13 (52.0)	χ² = 24.00	<0.001
Atypical vascular pattern, n (%)	5 (10.0)	13 (52.0)	χ² = 16.12	< 0.001
Multiple dermoscopic colors, n (%)	8 (16.0)	14 (56.0)	χ² = 12.86	< 0.001

Histopathological examination showed that 50 (66.7%) lesions were benign and 25 (33.3%) were malignant. Benign melanocytic nevus was the most common diagnosis, occurring in 25 (33.3%) patients, followed by seborrheic keratosis in 15 (20.0%) and dysplastic nevus in 10 (13.3%). Among the malignant lesions, basal cell carcinoma was diagnosed in 11 (14.7%) patients, malignant melanoma in eight (10.7%), and squamous cell carcinoma in six (8.0%) (Table 3).

**Table 3:** Histopathological spectrum of pigmented skin lesions (n = 75)

Histopathological diagnosis	N (%)
Benign melanocytic nevus	25 (33.3)
Seborrheic keratosis	15 (20.0)
Dysplastic nevus	10 (13.3)
Basal cell carcinoma	11 (14.7)
Squamous cell carcinoma	6 (8.0)
Malignant melanoma	8 (10.7)
Total benign lesions	50 (66.7)
Total malignant lesions	25 (33.3)

Dermoscopy demonstrated superior diagnostic performance compared to clinical examination. Its sensitivity was 92.0%, specificity was 94.0%, PPV was 88.5%, NPV was 96.0%, and overall accuracy was 92.0%. In comparison, clinical examination showed a sensitivity of 80.0%, a specificity of 82.0%, a PPV of 69.0%, an NPV of 89.1%, and an overall accuracy of 81.3%. Dermoscopy showed a sensitivity of 92.0%, a specificity of 94.0%, a PPV of 88.5%, an NPV of 95.9%, and an overall diagnostic accuracy of 93.3%. Dermoscopy demonstrated better diagnostic performance than clinical examination across all measures, with an overall accuracy of 93.3% and stronger agreement with histopathology (κ = 0.84) (Table 4).

**Table 4:** Diagnostic accuracy of clinical examination and dermoscopy for detecting cutaneous malignancy

Diagnostic parameter	Clinical examination	Dermoscopy
Sensitivity, %	80	92
Specificity, %	82	94
Positive predictive value, %	69	88.5
Negative predictive value, %	89.1	95.9
Overall accuracy, %	81.3	93.3
Cohen’s kappa	0.62	0.84

Dermoscopy demonstrated better diagnostic performance than clinical examination across all evaluated measures. Its sensitivity was 92.0% (95% CI: 75.0-97.8) compared to 80.0% (95% CI: 60.9-91.1) for clinical examination, while specificity was 94.0% (95% CI: 83.8-97.9) compared to 82.0% (95% CI: 69.2-90.2), respectively. Dermoscopy also showed a higher PPV (88.5% vs. 69.0%), a higher NPV (95.9% vs. 89.1%), and higher overall diagnostic accuracy (93.3% vs. 81.3%) (Table 5).

**Table 5:** Diagnostic performance of clinical examination and dermoscopy against histopathology for detection of cutaneous malignancy CI: confidence interval

Diagnostic measure	Clinical examination, % (95% CI)	Dermoscopy, % (95% CI)
Sensitivity	80.0 (60.9–91.1)	92.0 (75.0–97.8)
Specificity	82.0 (69.2–90.2)	94.0 (83.8–97.9)
Positive predictive value	69.0 (50.8–82.7)	88.5 (71.0–96.0)
Negative predictive value	89.1 (77.0–95.3)	95.9 (86.3–98.9)
Overall accuracy	81.3 (71.1–88.5)	93.3 (85.3–97.1)
Cohen’s kappa	0.62	0.84

## Discussion

In the present study, the correlation among clinical examination, dermoscopy, and histopathology in the evaluation of pigmented skin lesions for the early detection of cutaneous malignancies was evaluated. The mean age of the study population was 46.8 ± 15.2 years, with the majority in the 46-60 age group (32.0%). Men comprised 54.7% of the respondents, and over half reported being exposed to excessive sun (52.0%), while just 12.0% reported a family history of skin cancer. The lesions had a mean duration of 13.6 ± 8.9 months, and the mean diameter of the lesions was 9.8 ± 4.3 mm. These results corroborate those of previous studies, which found that pigmented skin lesions and skin malignancies are more common in middle-aged and elderly individuals, and that cumulative exposure to UV radiation is one of the most important risk factors for malignant transformation [18].

Histopathological findings revealed that two-thirds of the lesions were benign (66.7%) and one-third (33.3%) were malignant. Benign melanocytic nevus (33.3%) was the most common diagnosis, followed by seborrheic keratosis (20.0%) and dysplastic nevus (13.3%). Among the malignant lesions, basal cell carcinoma (14.7%), malignant melanoma (10.7%), and squamous cell carcinoma (8.0%) were the most frequent. These results illustrate the spectrum of pigmented lesions seen in routine tertiary dermatology practice. Previous studies have reported basal cell carcinoma as a common cutaneous malignancy, whereas melanocytic nevi represent a benign pigmented lesion; melanoma, although less frequent, remains an important malignant pigmented lesion because of its aggressive clinical behavior [19].

The present study showed that malignant lesions had significantly more suspicious clinical and dermoscopic features than the benign lesions. Malignant lesions were larger than benign lesions (13.7 ± 4.1 mm vs. 7.8 ± 2.6 mm, respectively; p < 0.001). Irregular borders, color variegation, asymmetry, blue-white veil, atypical vascular patterns, and multiple colors were all found at significantly higher rates in malignant lesions. These results confirm the diagnostic value of these dermoscopic features in the diagnosis of skin cancers. Previous evidence has demonstrated that dermoscopic features such as irregular pigmentation, blue-white veil, atypical pigment network, atypical vascular structures, streaks, and multicomponent patterns are significantly associated with melanoma and contribute to its dermoscopic recognition [20].

The sensitivity, specificity, PPV, NPV, and overall diagnostic accuracy of dermoscopy were 92.0%, 94.0%, 88.5%, 95.9%, and 93.3%, respectively. In comparison, clinical examination showed a sensitivity of 80.0%, a specificity of 82.0%, a PPV of 69.0%, an NPV of 89.1%, and an overall diagnostic accuracy of 81.3%. In addition, agreement between dermoscopy and histopathological diagnosis was considerably stronger (Cohen's kappa coefficient, 0.84) than that observed for clinical examination (Cohen's kappa coefficient, 0.62). These results suggest that dermoscopy improves diagnostic accuracy and decreases the number of missed malignancies and unnecessary biopsies performed.

Previous studies have also confirmed that dermoscopy has high diagnostic sensitivity and specificity compared to naked-eye examination, and demonstrates strong agreement with histopathological results when diagnosing melanoma subtypes [21]. The clinicodermoscopic-histopathological correlation observed in this study demonstrates the relevance of combining these two methods in clinical practice. Although clinical examination remains crucial for initial evaluation, dermoscopy is a valuable tool for increasing the accuracy of lesion characterization prior to biopsy, while histopathology remains the gold standard for diagnosis. Earlier studies have also found that dermoscopy is a useful tool for the quick diagnosis of skin cancers, enabling earlier and more accurate diagnosis than clinical examination alone [22].

This study has several limitations. It was conducted at a single tertiary-care center with a relatively small sample size of 75 patients, which may limit the generalizability of the findings. Selection bias is possible because only patients with clinically suspicious pigmented lesions who underwent biopsy were included. Dermoscopic assessment was performed after clinical examination; therefore, prior knowledge of the clinical appearance may have influenced dermoscopic interpretation and introduced sequential interpretation bias. Interobserver variability among clinicians, dermoscopists, and histopathologists was not assessed. In addition, standardized clinical, dermoscopic, and histopathological photographs were not systematically archived, which prevented independent visual verification of the reported clinicodermoscopic-histopathological correlations. Long-term follow-up and clinical outcomes were also not evaluated. Larger, multicenter, prospective studies with standardized image documentation, blinded assessments, and evaluation of interobserver agreement are recommended to confirm these findings.

In the present study, the correlation among clinical examination, dermoscopy, and histopathology in the evaluation of pigmented skin lesions for the early detection of cutaneous malignancies was evaluated. The mean age of the study population was 46.8 ± 15.2 years, with the majority in the 46-60 age group (32.0%). Men comprised 54.7% of the respondents, and over half reported being exposed to excessive sun (52.0%), while just 12.0% reported a family history of skin cancer. The lesions had a mean duration of 13.6 ± 8.9 months, and the mean diameter of the lesions was 9.8 ± 4.3 mm. These results corroborate those of previous studies, which found that pigmented skin lesions and skin malignancies are more common in middle-aged and elderly individuals, and that cumulative exposure to UV radiation is one of the most important risk factors for malignant transformation [18].

Histopathological findings revealed that two-thirds of the lesions were benign (66.7%) and one-third (33.3%) were malignant. Benign melanocytic nevus (33.3%) was the most common diagnosis, followed by seborrheic keratosis (20.0%) and dysplastic nevus (13.3%). Among the malignant lesions, basal cell carcinoma (14.7%), malignant melanoma (10.7%), and squamous cell carcinoma (8.0%) were the most frequent. These results illustrate the spectrum of pigmented lesions seen in routine tertiary dermatology practice. Previous studies have reported basal cell carcinoma as a common cutaneous malignancy, whereas melanocytic nevi represent a benign pigmented lesion; melanoma, although less frequent, remains an important malignant pigmented lesion because of its aggressive clinical behavior [19].

The present study showed that malignant lesions had significantly more suspicious clinical and dermoscopic features than the benign lesions. Malignant lesions were larger than benign lesions (13.7 ± 4.1 mm vs. 7.8 ± 2.6 mm, respectively; p < 0.001). Irregular borders, color variegation, asymmetry, blue-white veil, atypical vascular patterns, and multiple colors were all found at significantly higher rates in malignant lesions. These results confirm the diagnostic value of these dermoscopic features in the diagnosis of skin cancers. Previous evidence has demonstrated that dermoscopic features such as irregular pigmentation, blue-white veil, atypical pigment network, atypical vascular structures, streaks, and multicomponent patterns are significantly associated with melanoma and contribute to its dermoscopic recognition [20].

The sensitivity, specificity, PPV, NPV, and overall diagnostic accuracy of dermoscopy were 92.0%, 94.0%, 88.5%, 95.9%, and 93.3%, respectively. In comparison, clinical examination showed a sensitivity of 80.0%, a specificity of 82.0%, a PPV of 69.0%, an NPV of 89.1%, and an overall diagnostic accuracy of 81.3%. In addition, agreement between dermoscopy and histopathological diagnosis was considerably stronger (Cohen's kappa coefficient: 0.84) than that observed for clinical examination (Cohen's kappa coefficient: 0.62). These results suggest that dermoscopy improves diagnostic accuracy and decreases the number of missed malignancies and unnecessary biopsies performed.

Previous studies have also confirmed that dermoscopy has high diagnostic sensitivity and specificity compared to naked-eye examination, and demonstrates strong agreement with histopathological results when diagnosing melanoma subtypes [21]. The clinicodermoscopic-histopathological correlation observed in this study demonstrates the relevance of combining these two methods in clinical practice. Although clinical examination remains crucial for initial evaluation, dermoscopy is a valuable tool for increasing the accuracy of lesion characterization prior to biopsy, while histopathology remains the gold standard for diagnosis. Earlier studies have also found that dermoscopy is a useful tool for the quick diagnosis of skin cancers, enabling earlier and more accurate diagnosis than clinical examination alone [22].

This study has several limitations. It was conducted at a single tertiary-care center with a relatively small sample size of 75 patients, which may limit the generalizability of the findings. Selection bias is possible because only patients with clinically suspicious pigmented lesions who underwent biopsy were included. Dermoscopic assessment was performed after clinical examination; therefore, prior knowledge of the clinical appearance may have influenced dermoscopic interpretation and introduced sequential interpretation bias. Interobserver variability among clinicians, dermoscopists, and histopathologists was not assessed. In addition, standardized clinical, dermoscopic, and histopathological photographs were not systematically archived, which prevented independent visual verification of the reported clinicodermoscopic-histopathological correlations. Long-term follow-up and clinical outcomes were also not evaluated. Larger, multicenter, prospective studies with standardized image documentation, blinded assessments, and evaluation of interobserver agreement are recommended to confirm these findings.

## Conclusions

Dermoscopy showed higher diagnostic accuracy and stronger agreement with histopathology than routine clinical examination in the evaluation of pigmented skin lesions. It was particularly useful for improving the recognition of malignant lesions while maintaining high specificity. However, dermoscopy should be used as an adjunct to clinical assessment rather than a replacement for histopathological confirmation. Larger, multicenter studies with standardized image documentation and blinded assessments are needed to further validate these findings.
